# High fat induces activation of the tryptophan-ERK-CREB pathway and promotes bone absorption in cage layers

**DOI:** 10.1016/j.psj.2021.101149

**Published:** 2021-03-22

**Authors:** Jingying Ye, Xin Chi, Jinliang Wang, Zhiqiang Shen, Shu Li, Shiwen Xu

**Affiliations:** ⁎ Department of Veterinary Medicine, Northeast Agricultural University, Harbin 150030, PR China; † Shandong Binzhou Anim Sci and Vet Med Acad, Binzhou 256600, PR China

**Keywords:** cage layer fatigue, high fat diet, 5-HT-ERK-CREB, metabonomics

## Abstract

Cage layer fatigue is a common metabolic disease associated with a calcium and phosphorus imbalance, but recently we found this disease can be led by high fat diet. In order to elucidate the pathogenesis induced by a high fat diet, we randomly divided 88 White Shell Roman layers into 2 groups. There were 44 layers in each group. The control group was fed by a standard layer rations, and the high fat group was fed by completed rations mixing with 3% soybean oil. This study successfully constructed an animal model of osteoporosis caused by high fat. Bone samples were collected for bone mineral density, bone biomechanical properties which are all decreased at 26, 30, 34, and 38 wk old. We found the pathway of tryptophan-ERK-CREB from the perspective of metabonomics which promote the bone absorption. By metabolomics, we screened the significantly activated tryptophan pathway in high fat feed and detected the elevated tryptophan metabolite serum 5-HT at 26, 30, 34 and 38 wk old in the high fat group. At 38 wk old, we detected significantly elevated protein and mRNA levels of ERK/CREB/C-fos in bone tissue in the high fat group. So we concluded that high-fat were associated with a decrease in bone density and bone biomechanical index by disrupting tryptophan-5-HT-ERK1/2-CREB metabolism signaling pathways.

## INTRODUCTION

Osteoporosis, also known as cage layer fatigue in laying hens, is one of the most frequent bone diseases in modern large-scale laying facilities, and is also the most common fatigue comprehensive nutritional stress disease in raising chickens (Salai et al., 2000). A survey of cage layers in the UK showed that 29 % of them had fractures (Fleming, 2006). In 1955, Couch first proposed that brittleness increase, paralysis and death would occur in cage laying hens, and the disease was named CLF (Song, 2020). Recent studies have shown that there is a close and complex relationship between energy and bone metabolism, and excessive fat intake will cause bone metabolism diseases (Ayaki et al., 2019). Lavet et al. concluded that rats fed with high-fat diet showed reduced bone surface area, poorer bone thickness, and increased porosity of the tibial cortex (Raskin et al., 2010). Patsch et al. also found that the bone density and biomechanical indexes decreased in obese mice on a high-fat diet (Jiang et al., 2015; Ugarkovic, 2015). Myller et al. (2016) found that high-fat feeding seriously affected the economic benefit of laying hens in the breeding industry.

Fat has been found to cause pathological changes in bone tissue to some extent. Morphological analysis of bone tissue showed fat cavitation in the bone marrow of obese mice (Meier et al., 2010; McCormick et al., 2015). It's been detected experimentally increased adipocyte differentiation inhibit osteoblasts differentiation, and stimulated the inflammatory state (Xu and Komvopoulos, 2019). Similarly, Ryuichi et al. (2010) conducted a study to measure the bone marrow fat content of obese subjects, he considered fat metabolites stimulated osteoclasts to degrade bone tissue. Chattopadhyay et al., 2015 showed that triglyceride plays an important role in the occurrence of osteoporosis by stimulating osteoclasts generation. This suggests that high fat may play an important role in the development of osteoporosis, and we confirmed this in our study by constructing a model of osteoporosis in laying hens fed high fat.

The current research on this disease mainly focuses on the symptoms caused by the imbalance of calcium and phosphorus ratio in the diet. High fat daily food is also one of the causes of cage layer fatigue. So far, studies on high-fat diets have focused on mammals rather than poultry. Therefore, our study is about the relationship between high fat in the cage layer and osteoporosis.

## MATERIALS AND METHODS

### Animals Model

The study was conducted under the guidelines approved by the Animal Care and Use Committee of the Northeast Agricultural University, China. Ethics approval for all procedures to be carried out was obtained from Committee for Ethics in Research of the Northeast Agricultural University, China (No. 2017YFD0502206). This experimental animal White Shell Roman layer was purchased from Harbin's chicken farm. Eighty-eight 21-week-old White Shell Roman layers were randomly divided into 2 groups. The control group was fed with standard layer rations, and the high fat group was fed standard layer rations mixing with 3% soybean oil. Blood and tibia were collected from laying hens at 26, 30, 34, and 38 wk old.

### Measurement of Bone Index and Bone Length

Defrosted the tibia overnight and all soft tissue was scraped from the bone. Weight of each bone and its length was measured with a cursor caliper. Bone index formula = bone weight(g)/body weight(kg).

### Measurement of Bone Density

After removing the bone sample from the -80°C refrigerator and placing 30 min at room temperature, the dual energy x-ray was used for bone density determination. First, the model was placed on the instrument measurement plate for calibration. Then the bone sample was placed on the inspection table to determine the bone density of the middle of the femur. The numerical value is obtained by using the bone density measurement software.

### Biomechanics Measurement

Bone strength will be measured using a three-point bending test: Mark the midpoint of each bone and measure the diameter of the midpoint with a cursor caliper. According to the bone length of each bone, the span is determined. And the bone is placed on 2 fulcrum points so that the bone can be placed smoothly on the working platform. Preload speed set to 3 mm/min, stop when bone breaks.

### HE Staining

Bone tissue blocks were fixed in 10% formalin phosphate buffer solution for histomorphological detection: The above completely fixed tissues were fixed with 4% formaldehyde for 48 h, washed with tap water for 24 h, and then decalcified with decalcification solution. After the fixed tissue was repaired, the running water was rinsed for 4 h. Then, the tissue blocks were dehydrated with gradient alcohol, placed in xylene for 30 min and waxed for 2 h. After cooling, we slice the wax into 5 m slices on a slicer. Pick up and drop off the dry after biopsy in xylene Ⅰ Ⅱ soaked in 5 min, immerse 3 min each in gradient alcohol and distilled water, hematoxylin staining 5 min. Eosin staining solution for 3 min, finally to add just the right amount of neutral gum tissue biopsies, dried at room temperature; The staining effect was observed under a microscope then photograph under 200 × mirror.

### Total RNA Extraction and Quantitative Real-Time PCR (qPCR)

In the study total RNA was isolated using Trizol reagent, according to the manufacturer's instructions (Invitrogen, Shanghai, China). The dried RNA pellets were re-suspended in 50μl of water. The concentration and purity of the total RNA were determined spectrophotometrically at 260/280 nm. First-strand complementary DNA (**cDNA**) was synthesized from 5μg of total RNA using oligo dT primers and Superscript II reverse transcriptase according to the manufacturer's instructions (Invitrogen, Shanghai, China). Synthesized cDNA was diluted 5 times with sterile water and stored at −80°C, till further use (Chi et al., 2018).

Primer Premier Software 5.0 (PREMIER Biosoft International, USA) was used to design gene-special primers used for qPCR. β-actin was used as an internal reference. qPCR reactions were run in a 10 µL reaction mixture using a Light Cycler 480 II Detection System (Roche) Detection System (Applied Biosystems, USA). The gene sequence used is shown in Table 1.

### Western Blotting

Total protein was analyzed using mini gel sodium dodecyl sulfate polyacrylamide gel electrophoresis (**SDS-PAGE**) (12% acrylamide, 0.12% bisacrylamide, 30 min at 80 V plus 90 min at 120 V). Separated proteins were then transferred to PVDF membranes using a tank transfer for 2 h at 200 mA in Tris–glycine buffer containing 20% methanol. Membranes were blocked with 5% skim milk for 2 h at 37°C and incubated with diluted primary polyclonal antibodies. To remove surplus antibodies, the membranes were rinsed in PBST for 5 min. After 2 h of incubation with a horseradish peroxidase (**HRP**)-conjugated secondary antibody. The signal was detected on X-ray film (Trans Gen Biotech Co., China). The Image J system was used to detect the optical density (**OD**) of each band. Relative optical densities (arbitrary units) were obtained by normalizing each band for the GAPDH ban (Chi et al., 2018).

### Detection of Serum 5-HT

The blood naturally coagulates for 10 to 20 min, centrifugation for about 20 min, and carefully collects the supernatant. ELASA kit is used to detect serum 5-HT levels. 50 uL of diluted standard and biotin antigen working solution was added to the standard wells. 50 uL of sample and biotin antigen working solution was added to the sample wells. Gently shake, cover with sealing film, incubate for 30min in an incubator at 37°C, then wash. Then we add 50 uL of avidin -HRP into standard and sample wells and cover with sealing film, incubate at 37°C for 30 min, then wash. Then add 50 uL of reagent A to each hole, then add 50 uL of reagent B, gently shake and mix for 10 min. 50 uL of termination solution was added to each well to terminate the reaction. The absorbance of each hole was measured sequentially with the wave length of 450 nm and the blank hole was set to zero. The regression equation of the standard curve was calculated according to the concentration and OD value (Wang et al., 2019).

### Statistical Analysis

GraphPad Prism 7 was used for the statistical analysis and graph generation. The 2-way ANOVA was used to evaluate the data between the control group and the high fat group and are represented as means ± standard deviation (**SD**). *P* value (*P* < 0.05 [*], *P* < 0.01[**]) is used to represent the significant differences.

## RESULTS

### Effect of High Fat Diet on Tibia Density of Layers

**Figure 1.**
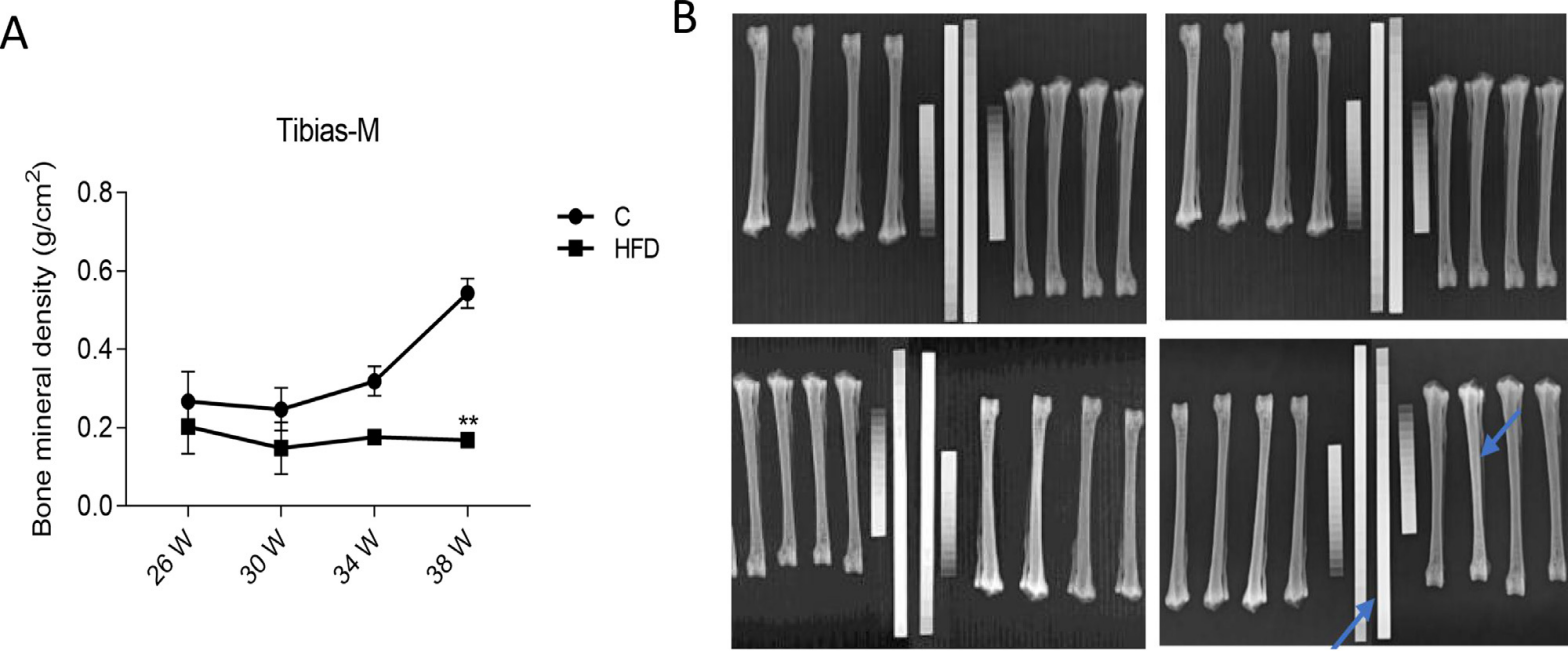
(A) The middle tibia density of cage layers. (B) The tibia X-ray film of cage layers at 38 wk old. The left figures represent the control group, the right figures represent the high fat group. Each value represents the mean ± SD. In each line, the line with “**” represent extremely significant differences between the groups (*P* < 0.01), and the line with “*” are significantly different (*P* < 0.05).

As shown in Figure 1, the mineral tibia density of control group in the whole trial period presents the general trend of increasing. But the density of tibia in high fat diet group down to the minimum 0.1478 g/cm^2^ from 30 wk old, which is stable in 0.16-0.17 g/cm^2^ later. The middle tibia mineral density of high fat group decreased obviously compared with the control group at 38 wk old (*P* < 0.01) (Figure 1A). As can be seen, the blue arrow corresponds to the bone density of the high-fat diet group which is lower than the control group, indicating that the feeding of the high-fat diet will have a certain influence on the bone density of layers (Figure 1B).

### Effect of High Fat Diet on Bone Length and Bone Index of Tibias

**Figure 2.**
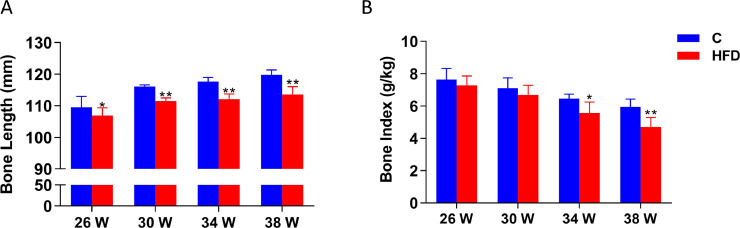
(A) The bone length of cage layers. (B) The bone index of cage layers of cage layers. Each value represents the mean ± SD. In each histogram, the bars with “**” represent extremely significant differences between the groups (*P* < 0.01), and the bars with “*”are significantly different (*P* < 0.05).

From Figure 2, the length from 30 to 38 wk old showed that the high fat group was significantly lower than the control group (*P* < 0.01) (Figure 2A). With the increase of feeding, the tibia index of both groups showed the same trend of decline. The tibia index from 34 and 38 wk old hens in high fat diet group were significantly lower than the control group, especially at 38 wk of age compared to the control (*P* < 0.01) (Figure 2B). These results indicate that long-term feeding of high fat diet appears to participate in bone metabolism and affect the development of bone.

### Effects of High Fat Diets on Biomechanical Properties of Tibias

**Figure 3.**
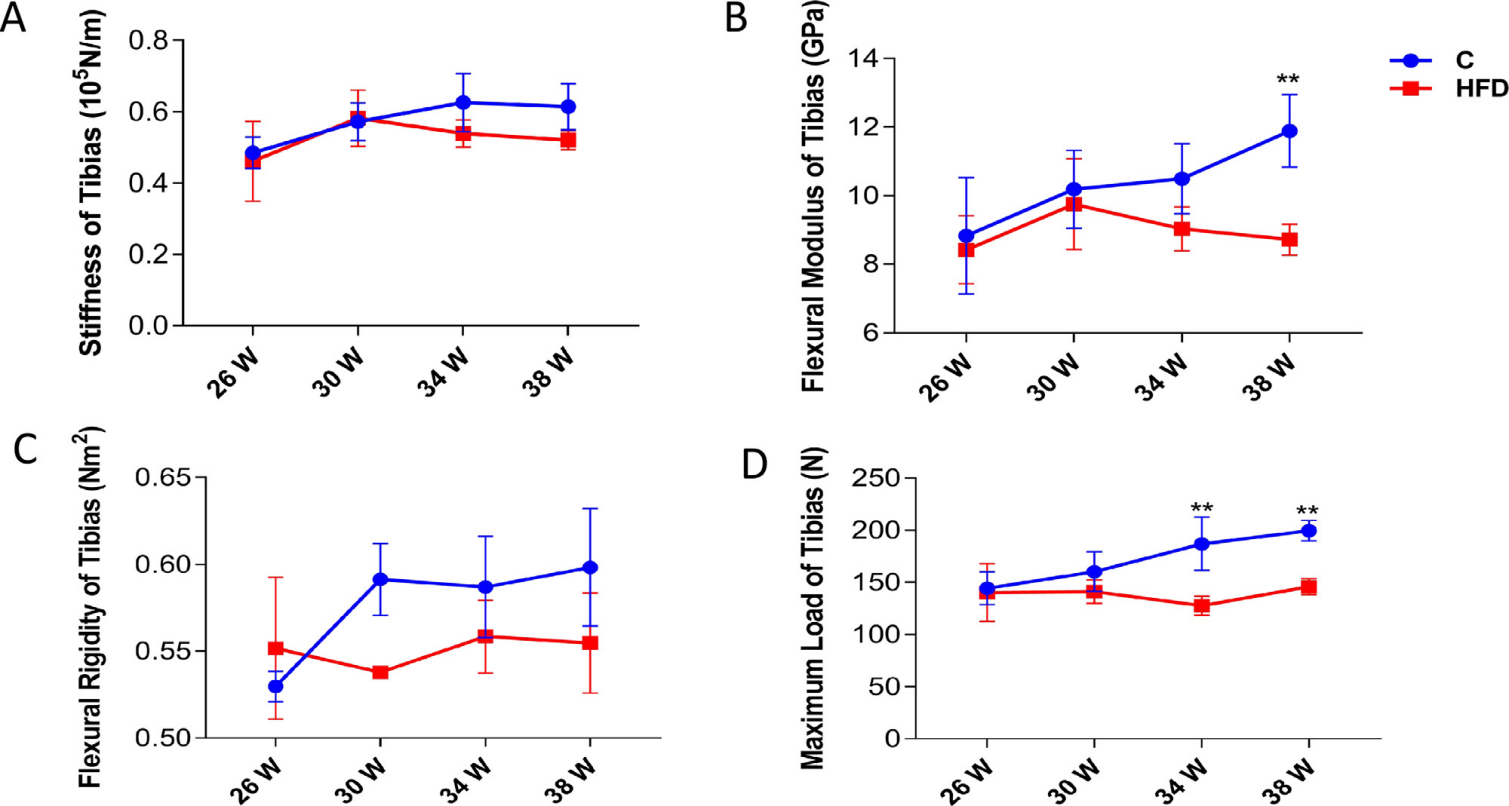
The biomechanics determination of cage layers. Each value represents the mean ± SD. In each histogram, the bars with “**” represent extremely significant differences between the groups (*P* < 0.01), and the bars with “*” are significantly different (*P* < 0.05). (A) Stiffness of tibia; (B) Flexural modules of tibia; (C) Flexural rigidity of tibia; (D) Maximum load level of tibia;

From the Figure 3, at 38 wk age, the flexural module of tibia in high fat diet group was significantly lower than the control group (*P* < 0.01) (Figure 3B). Except for 26 wk old, the flexural rigidity and stiffness in all high-fat diet groups was lower than the control group at the same time point, but there was no statistical difference between groups (Figure 3A, C). The results showed that the maximum load of tibia decreased significantly and the difference was significant at the age of 34 to 38 wk (*P* < 0.01) (Figure 3D). The results show that the flexural module, flexural rigidity, maximum load and stiffness in laying hens can be changed by feeding high fat diet for a long time.

### Effects of High Fat Diet on Bone Microstructure

**Figure 4.**
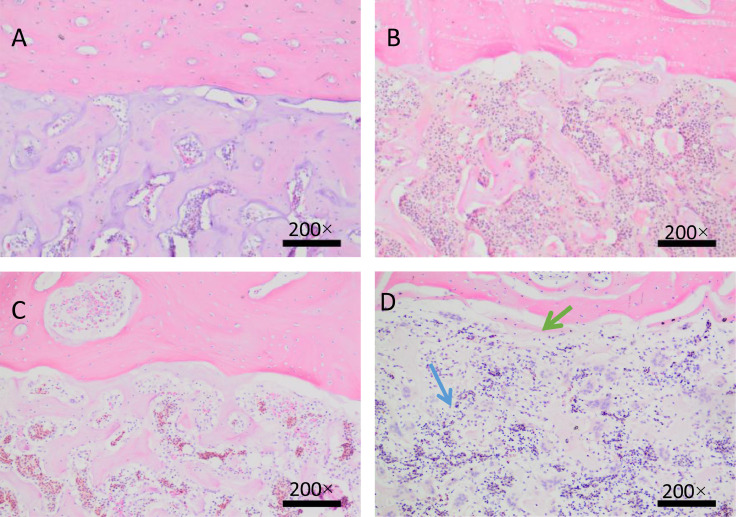
H&E staining of tibia tissues. The images are shown in 200× magnification. (A, C). Tibia of control group; (B, D). Tibia of high fat diet group.

From the Figure 4, the tibial cortex of laying hens in the control group had compact bone structure and compact bone trabecular. There was no obvious damage to the contact part between cortical bone and cancellous bone (Figure 4A, B). With the extension of the test time, at the later stage of the test at the age of 38 wk, the tibia of layers in the high-fat group was significantly damaged at the junction of cortical bone and medullary bone, and the spacing increased (red arrow) (Figure 4D). The bone trabeculae became thinner and thinner, the number decreased, the spacing increased and even the fracture occurred (blue arrow) (Figure 4D).

### Differential Metabolite Analysis and KEGG Enrichment Analysis

**Figure 5.**
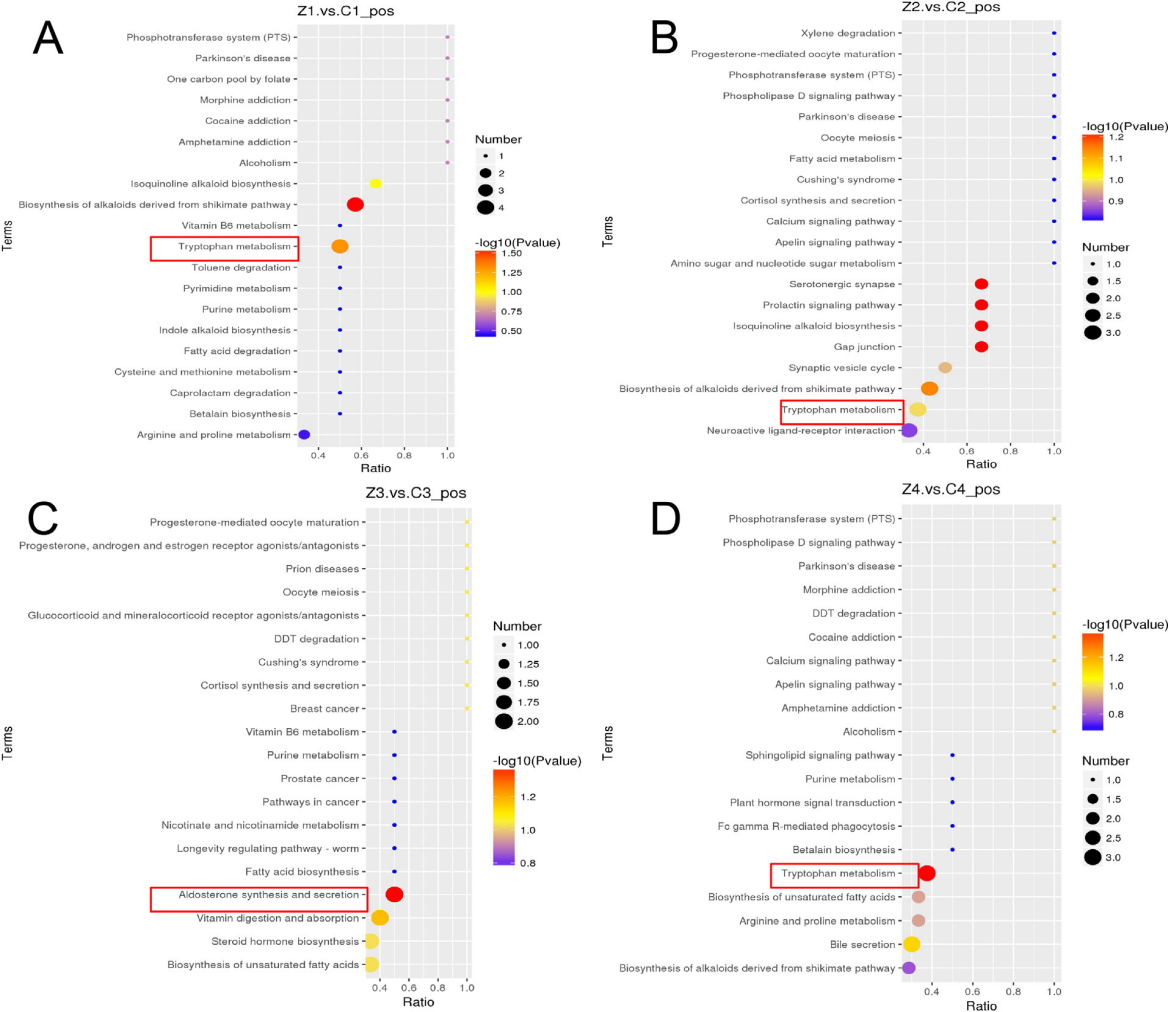
(A) Z1 vs. C1 positive ion KEGG enrichment bubble diagram; (B) Z2 vs. C2 positive ion KEGG enrichment bubble diagram; (C) Z3 vs. C3 positive ion KEGG enrichment bubble diagram; (D) Z4 vs. C4 positive ion KEGG enrichment bubble diagram; C1, C2, C3, C4 represent the control group at 26, 30, 34, 38 wk age respectively, Z1, Z2, Z3, Z4 represent the high fat group at 26, 30, 34, 38 wk age respectively. The abscissa in the figure is the ratio of the number of differential metabolites in the corresponding pathway to the total number of identified metabolites. The higher the value is, the higher the enrichment degree of differential metabolites in the pathway is. The color of the dot represents the *P*-value value of the hypergeometric test. The smaller the value is, the more reliable and statistically significant the test is. The size of the point represents the number of differential metabolites in the corresponding pathway, the larger the number of differential metabolites in the pathway.

From the Table 2, according to Variable Importance in the Projection, 1026 different metabolites were identified in serum positive ions between the high-fat group and the control group. The screening results of serum metabolites with significant differences in each group were the number of down-regulated compounds greater than the number of up-regulated compounds. According to the enrichment results, the bubble map of the enriched KEGG pathway was drawn, and the serum of layers in the high-fat group and the control group were detected by metabolites, and the differential metabolites were analyzed, and a total of 2 metabolic pathways were significantly activated: Tryptophan metabolism is significantly activated at 26, 30, 38 wk old; Aldosterone synthesis biosynthesis were significantly activated at 34 wk of age (Figure 5).

**Table 1:** mRNAs primer sequences.

Gene		Primer sequence (5′ → 3′)	Length of the primer (bp)
MEK	FORWARD	TAGTTCTGTAGCTCCCCGCT	20
	REVERSE	CACCTGGGACTGCGTTTTTG	
ERK	FORWARD	CAGGGACATCAAGCCGTGTT	20
	REVERSE	TGCCAGACCTCTAGTGAGGC	
CREB	FORWARD	CACCAGGCTGCGTCTCATCA	20
	REVERSE	TGCCAGACCTCTAGTGAGGC	
C-fos	FORWARD	ACGAGGAGAAATGCCTGACG	20
	REVERSE	CTTCAGATTGGCGAGGAGGG	
GAPDH	FORWARD	TCGGAGTGAACGGATTTGGC	20
	REVERSE	TGACAAGCTTCCCGTTCTCC

**Table 2:** The number of different metabolites up-regulated and down-regulated.

Compared samples	Num. of total ident.	Num. of total sig	Num. of sig. up	Num. of sig. down
Z1.vs.C1_pos	1026	212	58	154
Z2.vs.C2_pos	1026	205	64	141
Z3.vs.C3_pos	1026	81	32	49
Z4.vs.C4_pos	1026	134	48	86

### Results of High Fat Diet on Serum 5-HT Content and ERK/CREB mRNA and Protein Expression in Layers

**Figure 6.**
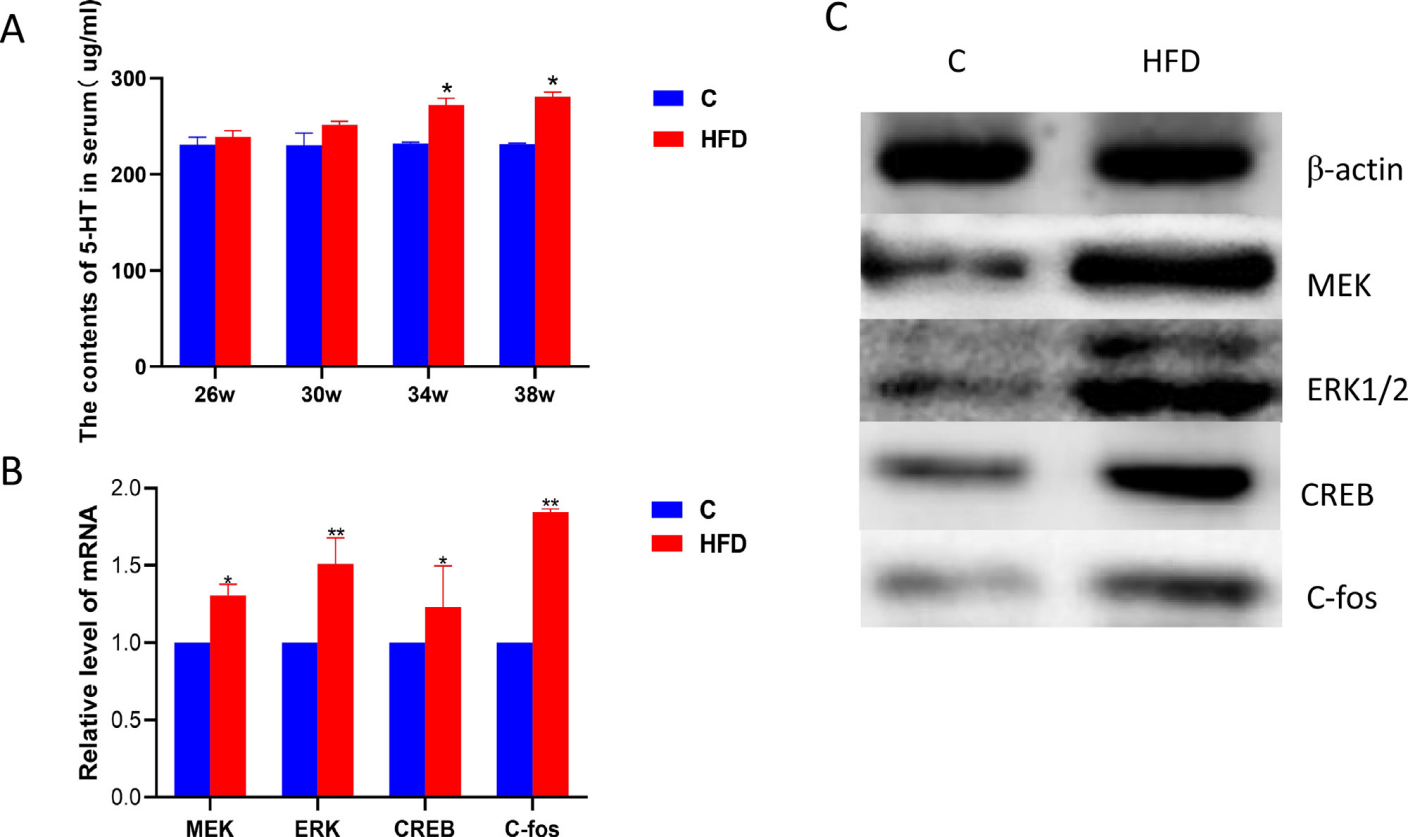
(A) The content of serum 5-HT of control and high fat feeding groups. (B) Relative mRNA of control and high fat feeding groups. (C) Relative protein of control and high fat feeding groups. Each value represents the mean ± SD. In each histogram, the bars with “**” represent extremely significant differences between the groups (*P* < 0.01), and the bars with “*”are significantly different (*P* < 0.05).

The Figure 6 shows the effect of high fat diets on expression associated with the 5-HT/ERK/CREB pathway. At 34 and 38 wk old, the serum 5-HT was significantly higher than the control (*P* < 0.05) (Figure 6A). The relative mRNA levels of MEK, CREB were similar in the 4 groups. Their expression in the high fat group was higher than in the control group (*P* < 0.05) at 38 wk old. In the C-fos gene expression level, the expression level of the high fat group was 1.9 times that of the control group, the difference was statistically significant (*P* < 0.01) (Figure 6B). And their protein expression showed the same results (Figure 6C).

## DISCUSSION

Our study has shown that layer has fed by long-term fat can develop osteoporosis and be a model for study. Dual energy X-ray absorptiometry, which assayed bone density and femoral mineral salt content, is commonly used to detect bone health (Cusano et al., 2016). In this study, the tibia density was significantly reduced after 38 wk of feeding a high-fat diet, and the tibial cortex was partially loose. Bone biomechanics is also a reliable method for evaluating bone quality, which is to study the mechanical characteristics of bone tissue under external action and the biological effects of bone under stress. At present, biomechanical testing has become the “Gold Standard” for evaluating biomechanical models of osteoporosis (Fyhrie and Vashishth, 2000). Fracture is closely related to the biomechanical properties of bone (Salai et al., 2000). The experiments of flexural rigidity and stiffness can not only reflect the biomechanical condition of bone, but also help to evaluate the incidence of fractures (Zhang and Ren, 2016). We found the flexural rigidity and stiffness in all high-fat diet groups was lower than the control group at the same time point. The results showed that the maximum load of tibia decreased significantly at the end of trial, also showed that the biomechanical level can be changed by feeding high fat diet for a long time.

Our metabonomics data showed that the tryptophan metabolic pathway was significantly activated in the high-fat group at 26, 30 and 38 wk old. Min proposed that the pathogenesis of obesity is related to the disturbance of tryptophan and multiple leg diseases (Kim and Bae, 2013). Activation of the tryptophan pathway produces 5-HT, which is one of the neurotransmitters in bone (Ducy and Karsenty, 2010). In 1941 Rapport et al isolated 5-HT from serum (Ducy, 2011). 5-HT is associated with diet, particularly with high fat intaking (Vijay and Yadav, 2018). If the level of 5-HT in blood is increased, cortical bone thickness is significantly reduced (Sampson and Mazmanian, 2015). Recent studies have found that endogenous 5-HT level plays an important negative regulatory role on bone metabolism through targeting cell receptors in distant bone tissue (Gershon and Tack, 2007; Mödder et al., 2010). Several 5-HT receptors express higher during the osteoclast differentiation (Sánchez-Duffhuesa and Hiepen, 2015). The binding of enteric 5-HT to its receptor 5-HT1BR on osteoblasts can also inhibit the proliferation of osteoblasts (Segula, 2014). Yadav et al. (2010) found that the number of osteoblasts increased and the phenotype of high bone mass appeared in HTR1B - / - mice. Chabbi et al. found that inhibition of 5-HT synthesis may be a way to enhance bone anabolism and thus treat osteoporosis (Chabbi-Achengli et al., 2012; Jean-Marie and Philippe, 2012). In this experiment, the content of 5-HT in serum of 38-week-old high-fat-feeding laying hens was significantly higher than the control, proving that 5-HT generated by tryptophan pathway was related to bone resorption.

5-HT signaling pathway can induce osteoporosis and participate in bone metabolism through potential candidate genes encoding bone metabolism (Stuart, 2010). This experiment found that 5-HT induced activation of the downstream ERK1/2 pathway. ERK1/2 is the most abundant P38 member in bone and marrow, and plays an important role in physiological bone homeostasis. ERK1 and ERK2 play an important role in osteoclast formation, differentiation and stimulation of bone resorption (Bienvenu et al., 2015). ERKs activate the SRE in the nucleus and regulate the C-fos gene transcription, thereby transforming mature macrophages into osteoclast. C-fos protein expression leads to the change of bone remodeling microenvironment, thus leading to the occurrence of primary osteoporosis (Takayanagi et al., 2012). Liu et al. found that under physiological synaptic stimulation of 5-HT, ERK-CREB pathway was slowly activated, thus promoting osteoclast differentiation (Liu et al., 2016). Experimental results also showed that ERK1-/- bone marrow polymorphonuclear cell transplantation significantly increased bone mineral density in mice (Lonze and Ginty, 2002). In this study, the expressions of MEK, ERK1/2 and CREB in the high-fat group were all higher than the control. Thus, activation of the tryptophan pathway has been shown to activate 5-HT/ERK/CREB for bone resorption.

The high incidence of fracture indicates that osteoporosis is one of the welfare problems in chickens. The current research on this disease mainly focuses on the symptoms caused by the imbalance of calcium and phosphorus ratio in the diet. High fat diet is also one of the causes of cage layer fatigue. Therefore, an in-depth understanding of the molecular mechanism of obesity-induced osteoporosis is helpful to provide necessary theoretical reference for the prevention and treatment of osteoporosis.

## CONCLUSIONS

High-fat layers were associated with a decrease in bone density and a decrease in bone biomechanical index. And high fat may participate in bone metabolism by disrupting tryptophan-5-HT-ERK1/2-CREB metabolism signaling pathways.
